# Three New Species of *Hypoxylon* (Xylariales, Ascomycota) on a Multigene Phylogeny from Medog in Southwest China

**DOI:** 10.3390/jof8050500

**Published:** 2022-05-11

**Authors:** Zi-Kun Song, An-Hong Zhu, Zhen-Dong Liu, Zhi Qu, Yu Li, Hai-Xia Ma

**Affiliations:** 1 Institute of Tropical Bioscience and Biotechnology, Chinese Academy of Tropical Agricultural Sciences, Haikou 571101, China; michellesong2021@yeah.net (Z.-K.S.); quzhi@itbb.org.cn (Z.Q.); 2 College of Plant Protection, Jilin Agricultural University, Changchun 130118, China; liyu@itbb.org.cn; 3 Chinese Academy of Tropical Agricultural Sciences, Haikou 571101, China; 18289679317@163.com; 4 Food Science College, Tibet Agriculture & Animal Husbandry University, Nyingchi 860000, China; liuzhendong@xza.edu.cn; 5 Hainan Institute for Tropical Agricultural Resources, Haikou 571101, China; 6 Hainan Key Laboratory of Tropical Microbe Resources, Haikou 571101, China

**Keywords:** Ascomycota, *Hypoxylon*, multigene phylogeny, taxonomy, wood-decomposing fungi, Xylariales

## Abstract

During a survey of hypoxylaceous fungi in Medog county (Tibet Autonomous Region, China), three new species, including *Hypoxylon damuense*, *Hypoxylon medogense*, and *Hypoxylon zangii*, were described and illustrated based on morphological and multi-gene phylogenetic analyses. *Hypoxylon damuense* is characterized by its yellow-brown stromatal granules, light-brown to brown ascospores, and frequently indehiscent perispore. *Hypoxylon medogense* is morphologically and phylogenetically related to *H. erythrostroma* but differs in having larger ascospores with straight spore-length germ slit and conspicuously coil-like perispore ornamentation. *Hypoxylon zangii* shows morphological similarities to *H. texense* but differs in having Amber (47), Fulvous (43) and Sienna (8) KOH-extractable pigments and larger ascospores with straight spore-length germ slit. The multi-gene phylogenetic analyses inferred from the datasets of ITS-RPB2-LSU-TUB2 supported the three new taxa as separate lineages within *Hypoxylon*. A key to all known *Hypoxylon* species from China and related species worldwide is provided.

## 1. Introduction

Polyphasic taxonomic studies based on phylogenetic, chemotaxonomic, and morphological data were extensively applied to identify species and reflect evolutionary relationships of hypoxylaceous fungi in recent years [1,2,3]. Since resurrected and emended by Wendt et al. [2], 15 genera were rearranged and recognized to Hypoxylaceae by having stromatal pigments and a nodulisporium-like anamorph. According to the arrangement of the families in Sordariomycetes by Hyde et al. [4], 19 genera were accepted in Hypoxylaceae as saprobes and endophytes. Interesting, *Hypoxylon* species in endophytic stages may play an important ecological role in protecting their host plants from pathogens [4], and some species are related to insect vectors [2,5,6,7]. As the main family of Xylariales, Hypoxylaceae exhibits high diversity in tropical and subtropical areas [8,9,10,11]. In the classification system of Ju and Rogers [12], the genus *Hypoxylon* Bull. contains two subclades, the *Annulata* and *Hypoxylon* sections. Then they were segregated and the *Annulata* section was accepted as a new genus, *Annulohypoxylon*, based on molecular phylogenetic data inferred from ACT and TUB2 sequences [13]. *Hypoxylon* species are mainly saprobic on dead and decaying wood of angiospermous plants [14]. In this genus, more than 200 species with 1189 epithets included in the Index Fungorum have been reported so far [4,15,16]. Despite species of *Hypoxylon* being widely distributed throughout Asia, only 57 species were reported in China currently [17,18,19,20,21].

Medog county, Tibet Autonomous Region is located in southwest China, at the eastern end of the Himalayas and the lower reaches of the Yarlung Zangbo River, and belongs to a subtropical humid climate zone in the Himalayas, with abundant rainfall and an average annual temperature of 18.0 °C [22]. These unique climatic conditions contribute to the abundant resources of macro-fungi. In the current study, we surveyed hypoxylaceous taxa in Medog county, and three undescribed species of *Hypoxylon* were identified. The morphological characteristics of the three new species were described, and their nucleotide sequences were analyzed phylogenetically to confirm their status within *Hypoxylon*.

## 2. Materials and Methods

### 2.1. Collection of Specimens

The studied specimens were collected from Medog county (Tibet Autonomous Region), which is located in southwestern China. The explored sites are approximately at elevations from 800 to 1600 m above sea level (m.a.s.l.). The collected samples were dried with a portable drier (manufactured in Germany). Dried samples were labeled and then stored by ultrafreezing at −80 °C for a week to kill insects and their eggs before they were ready for studies. The Fungarium of the Institute of Tropical Bioscience and Biotechnology, Chinese Academy of Tropical Agricultural Sciences (FCATAS) is responsible for the preservation of specimens.

### 2.2. Morphological Observations

Sexual structures of the collected specimens were used for morphological observations and identification. The stroma and perithecia were observed, photographed and measured with a VHX-600E 3D microscope from the Keyence Corporation (Osaka, Japan). Fresh material was respectively immersed in water, 10% KOH, and Melzer’s reagent to observe micromorphological structures as determined by Ma et al. and Song et al. [20,21]. The observations, micrographs, and measurements of asci and ascospores were performed by using an Olympus IX73 inverted fluorescence microscope (Olympus, Tokyo, Japan) and the CellSens Dimensions Software (Olympus, Tokyo, Japan). The observations and photographs of ornamentation of ascospores were examined by scanning electron microscope (SEM) (Phenom Corporation, Netherlands) as given in Friebes and Wendelin [23]. The stromatal color and KOH-extractable pigments were assigned following the mycological color chart of Rayner [24]. The present paper contains the following abbreviations: KOH = 10% potassium hydroxide; n = number of measuring objects; M = arithmetical average of sizes of all measuring objects.

### 2.3. DNA Extraction, Amplification, and Sequencing

Fresh tissue of stroma was used for DNA extraction and sequence generation following the suggestions by Ma et al. and Song et al. [20,21]. Sequences of four DNA loci—ITS (internal transcribed spacer regions), nrLSU (nuclear large subunit ribosomal DNA), RPB2 (RNA polymerase II second largest subunit), and β-tubulin (beta-tubulin) were selected for multi-gene phylogenetic analyses [2,25]. The target sequences were amplified by the primers ITS4/ITS5, LR0R/LR5, fRPB2-7CR/fRPB2-5F, and T1/T22 [26,27,28,29,30]. In total, six ITS, six LSU, six RPB2, and six β-tubulin sequences of new *Hypoxylon* specimens collected from Medog were obtained and submitted to GenBank.

### 2.4. Molecular Phylogenetic Analyses

The listed Hypoxylaceae and Xylariaceae species in Table 1 originated from previously published studies. Besides *Hypoxylon* spp., the backbone tree contained species of related genera including *Annulohypoxylon*, *Daldinia*, *Hypomontagnella*, *Jackrogersella*, *Pyrenopolyporus*, *Rhopalostroma*, and *Thamnomyces* with *Xylaria hypoxylon* (L.) Grev. and *Biscogniauxia nummularia* (Bull.) Kuntze chosen to be outgroups.

The alignment, trimming, and concatenation of sequences followed Song et al. [21]. The multi-gene phylogenetic analyses were performed by using two methods of maximum likelihood (ML) and Bayesian analyses (BA) based on ITS-LSU-RPB2-β-tubulin datasets and ITS-β-tubulin datasets. The latter was used for an added validation to the former. Maximum likelihood analyses used raxmlGUI 2.0 with 1000 bootstrap replicates and GTRGAMMA+G as a substitution model [20,31,32]. Bayesian analyses used MrBayes 3.2.6 with jModelTest 2 conducting model discrimination and Markov chain Monte Carlo (MCMC) sampling. Every 100th generation was sampled as a tree with 1,000,000 generations running for six MCMC chains [20,33]. Phylogenetic trees were viewed and edited by FigTree version 1.4.3 and Photoshop CS6.

This study selected 89 taxa from 10 genera to perform phylogenetic analysis, including 3 *Annulohypoxylon* spp., 2 *Daldinia* spp., 3 *Hypomontagnella* spp., 72 *Hypoxylon* spp*.,* 2 *Jackrogersella* spp., 3 *Pyrenopolyporus* spp., 1 *Rhopalostroma* sp., and 1 *Thamnomyces* sp. with *X*. *hypoxylon* and *B*. *nummularia* added as the outgroups. The sequence datasets comprised 306 sequences with 91 ITS, 62 LSU, 62 RPB2, and 91 β-tubulin sequences. After being aligned and trimmed, the combined dataset contained 3530 characters including gaps with 587 characters for ITS, 867 characters for LSU, 729 characters for RPB2, and 1347 characters for β-tubulin alignment, of which 1537 characters were parsimony-informative.

## 3. Results

### 3.1. Phylogenetic Analysis

**Figure 1:**
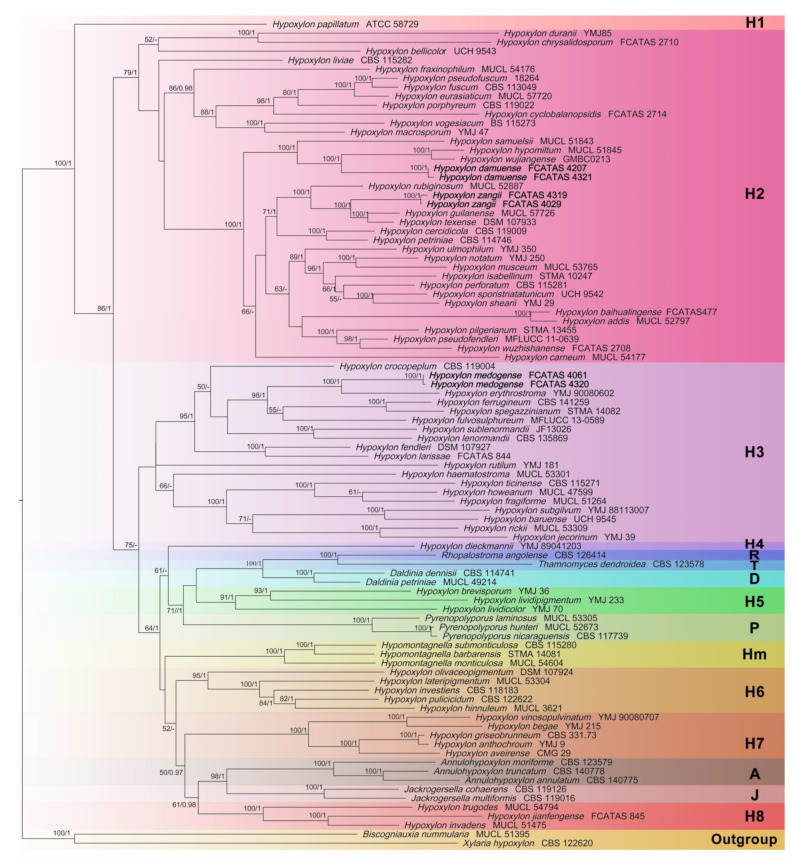
Phylogram of the best ML trees of the *Hypoxylon* species from an analysis based on multi-gene alignment of ITS-LSU-RPB2-β-tubulin. ML bootstrap support (BS) ≥ 50% and Bayesian posterior probabilities (PP) ≥ 0.95 are labelled above or below the respective branches (BS/PP). Species in **bold** were sequenced in this study.

The best-scoring ML tree was built with a final ML optimization likelihood value of −77,579.198447. Bayesian posterior probabilities were calculated with a final average standard deviation of split frequencies of less than 0.01. Phylogenetic trees of BA and ML analyses were found to be highly similar in topology, and the ML tree is represented in Figure 1. ML bootstrap support (BS) ≥ 50% and Bayesian posterior probabilities (PP) ≥ 0.95 were labelled along the branches, while branches with BS ≥ 70% and PP ≥ 0.98 were considered to be significant.

Multi-gene phylogeny shows that our new species are clustered within the clades H2 and H3. *Hypoxylon damuense* and *H. zangii* are phylogenetically well differentiated. *Hypoxylon damuense* clustered with *H*. *hypomiltum* Mont. and *H*. *wujiangense* Y.H. Pi, Q.R. Li in a full support subclade (BS = 100%, PP = 1) in clade H2. *Hypoxylon zangii* clustered together with *H*. *guilanense* Pourmogh., C. Lamb. and *H*. *texense* Kuhnert, Sir in a full support subclade as a sister to *H*. *rubiginosum* (Pers.) Fr. *Hypoxylon medogense* formed a subclade with *H*. *erythrostroma* J.H. Mill. with full support in clade H3. The phylogenetic tree shows that *Hypoxylon* is a paraphyletic group with other genera embedded (e.g., *Annulohypoxylon*, *Daldinia*, and *Hypomontagnella*).

### 3.2. Taxonomy

**Figure 2:**
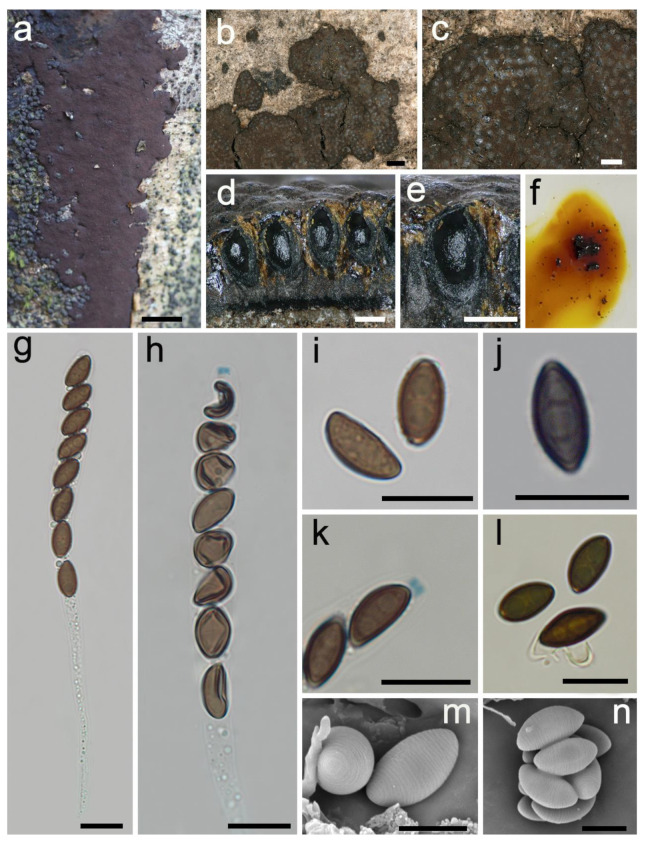
*Hypoxylon damuense* (holotype FCATAS 4207). (**a**,**b**) Stromata on the bark of dead wood. (**c**) Stromatal surface. (**d**,**e**) Stroma in vertical section showing perithecia and ostioles. (**f**) KOH-extractable pigments. (**g**) Asci in water. (**h**) Asci in Melzer’s reagent. (**i**) Ascospores in water. (**j**) Ascospore in 10% KOH showing germ slit. (**k**) Apical apparatus in Melzer’s reagent. (**l**) Ascospores in 10% KOH. (**m**,**n**) Ascospores under SEM. Scale bars: (**a**) = 1 cm; (**b**) = 1000 µm; (**c**) = 500 µm; (**d**,**e**) = 200 µm; (**g**–**l**) = 10 µm; (**m**,**n**) = 5 µm.

***Hypoxylon damuense*** Hai X. Ma, Z.K. Song and Y. Li, sp. nov., Figure 2.

MycoBank: MB 843581

**Diagnosis.** Differs from *H. rubiginosum* in its larger asci, light-brown to brown ascospores with conspicuous coil-like ornamentation and most of the perispore indehiscent. Differs from *H. hypomiltum* in its smaller perithecia, larger asci and apical apparatus. Differs from *H. wujiangense* in its larger stromata and stromatal KOH-extractable pigments.

**Etymology.** *Damuense* (Lat.): referring to the holotype locality of species in Damu Township.

**Holotype.** CHINA: Tibet Autonomous Region, Medog County, Damu Township, Kabu Village, 29°38′42″ N, 95°37′44″ E, alt. 1280 m, saprobic on the bark of dead wood, 2 October 2021, Haixia Ma, Col. XZ207 (FCATAS 4207).

**Teleomorph.** Stromata pulvinate to effused-pulvinate, 1–9 cm long × 0.4–2 cm broad × 0.6–0.9 mm thick; with inconspicuous to conspicuous perithecial mounds; surface Bay (6), Rust (39) and Livid Purple (81), exposing black subsurface layer when colored coating worn off; with yellow-brown granules immediately beneath the surface and between perithecia; yielding luteous (12) and ochreous (44) to fulvous (43) KOH-extractable pigments; tissue below the perithecial layer black, 0.1–0.46 mm thick. Perithecia ovoid, black, 0.16–0.3 mm broad × 0.3–0.45 mm high. Ostioles umbilicate, opening lower than the stromatal surface or at the same level as the stromatal surface. Asci cylindrical with eight obliquely uniseriate ascospores, long-stipitate, 102–242 µm total length, the spore-bearing portion 60–72 µm long × 6.2–8.6 µm broad, and stipes 41–174 µm long, with amyloid apical apparatus bluing in Melzer’s reagent, discoid, 0.8–1.5 µm high × 1.6–2.4 µm broad. Ascospores light-brown to brown, unicellular, ellipsoid-inequilateral, with narrowly rounded ends, 8.2–10.5 × 4.1–5.5 µm (n = 60, M = 9.2 × 4.8 µm), with straight spore-length germ slit on the convex side; most of the perispore indehiscent in 10% KOH, occasionally dehiscent, with conspicuous coil-like ornamentation in SEM; epispore smooth.

**Additional specimens examined.** CHINA: Tibet Autonomous Region, Medog County, Damu Township, Kabu Village, 29°38′48″ N, 95°37′46″ E, alt. 1310 m, saprobic on the bark of dead wood, 2 October 2021, Haixia Ma, Col. XZ321(FCATAS 4321).

**Note.** *Hypoxylon damuense* was found in the subtropics, and characterized by large pulvinate stromata, long asci stipes, amyloid apical apparatus, light-brown to brown ascospores with straight germ slit, most of the perispore indehiscent in 10% KOH, with conspicuous coil-like ornamentation. The new species is quite similar to *H. rubiginosum* in ascospore dimensions and KOH-extractable pigments, but the latter has darker colored ascospores, smaller asci (100–170 µm total length), dehiscent perispores and smooth or with inconspicuous coil-like ornamentation. *Hypoxylon rubiginosum sensu stricto* was always discovered in the temperate northern hemisphere except for samples reported in Florida [12,15,48]. Moreover, the status of *H. damuense* as a new species is also supported in the phylogenetic trees, where it appears distant from *H. rubiginosum*.

Although phylogenetic analyses showed that *H. damuense* clustered with *H. hypomiltum* and *H. wujiangense* in a clade with strong supported values (100%/1), there are distinct morphological differences among them. *Hypoxylon hypomiltum* differs in having larger perithecia ((0.2–)0.3–0.5 mm broad × 0.5–0.7 mm high), smaller asci (90–132(–145) µm total length), smaller apical apparatus (0.3–0.6 µm high × 1.2–1.5 µm broad) and slightly oblique to sigmoid germ slit [12]. *Hypoxylon wujiangense* can be distinguished by its smaller stromata with white pruina surface, Sienna (8) KOH-extractable pigments and larger apical apparatus 1.5–2 µm high × 2.5–3 µm broad [19].

**Figure 3:**
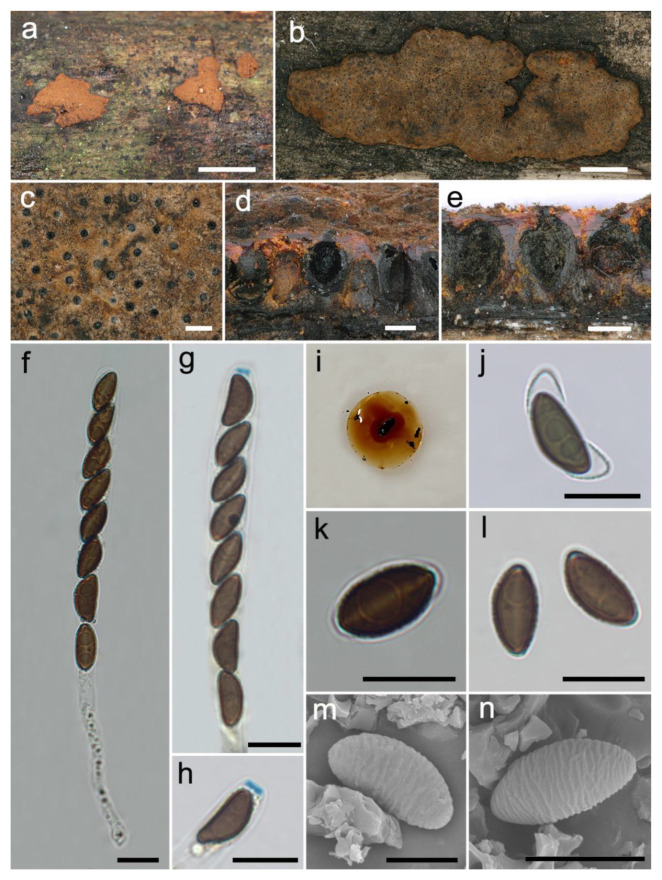
*Hypoxylon medogense* (holotype FCATAS 4061). (**a**,**b**) Stromata on the bark of dead wood. (**c**) Stromatal surface. (**d**,**e**) Stroma in vertical section showing perithecia and ostioles. (**f**) Asci in water. (**g**) Asci in Melzer’s reagent. (**h**) Apical apparatus in Melzer’s reagent. (**i**) KOH-extractable pigments. (**j**) Ascospore in 10% KOH. (**k**) Ascospore in water showing germ slit. (**l**) Ascospores in water. (**m**,**n**) Ascospore under SEM. Scale bars: (**a**) = 1 cm; (**b**) = 2 mm; (**c**–**e**) = 200 µm; (**f**–**h,j**–**l**) = 10 µm; (**m**) = 5 µm; (**n**) = 8 µm.

***Hypoxylon medogense*** Hai X. Ma, Z.K. Song and Y. Li, sp. nov., Figure 3.

MycoBank: MB 843582

**Diagnosis.** Differs from *H. erythrostroma* in its larger ascospores with straight spore-length germ slit and very conspicuous coil-like perispore ornamentation. Differs from *H.* *laschii* in ovoid to obovoid perithecia, shorter asci, and larger ascospores with very conspicuous coil-like perispore ornamentation.

**Etymology.** *Medogense* (Lat.): referring to the holotype locality of species in Medog county.

**Holotype.** CHINA: Tibet Autonomous Region, Medog County, Dexing Township, Deguo village, 29°24′58″ N, 95°23′6″ E, alt. 814 m, saprobic on the bark of dead wood, 25 September 2021, Haixia Ma, Col. XZ61 (FCATAS 4061).

**Teleomorph.** Stromata plane, pulvinate to effused-pulvinate, 3.9–16.5 cm long × 2.5–6.2 cm broad × 0.52–0.72 mm thick; with inconspicuous to conspicuous perithecial mounds; surface cinnamon (62), fulvous (43), ochreous (44) and bay (6); with orange or reddish-orange granules immediately beneath the surface and between perithecia; yielding amber (47), orange (7) or scarlet (5) KOH-extractable pigments; tissue below the perithecial layer inconspicuous, black. Perithecia ovoid to obovoid, black, 0.16–0.3 mm broad × 0.25–0.4 mm high. Ostioles with conical black papillae, opening higher than the stromatal surface. Asci cylindrical, eight-spored, uniseriate, 91–142 µm total length, the spore-bearing portion 60–79 µm long × 6.9–9.4 µm broad, and stipes 25–85 µm long, with amyloid apical apparatus bluing in Melzer’s reagent, discoid, 0.9–1.4 µm high × 2.4–2.9 µm broad. Ascospores brown to dark brown, unicellular, ellipsoid-inequilateral, with narrowly rounded ends, 9.9–12.8 × 4.6–7 µm (n = 60, M = 11.1 × 5.7 µm), with straight spore-length germ slit on the convex side; perispore dehiscent in 10% KOH, with very conspicuous coil-like ornamentation in SEM; epispore smooth.

**Additional specimens examined.** CHINA: Tibet Autonomous Region, Medog County, Dexing Township, Deguo village, 29°25′28″ N, 95°23′26″ E, alt. 808 m, saprobic on the bark of dead wood, 25 September 2021, Haixia Ma, Col. XZ320 (FCATAS 4320).

**Note.***Hypoxylon medogense* is characterized by having a bright orange red waxy layer beneath the surface, orange (7) or scarlet (5) KOH-extractable pigments, ostioles higher than the stromatal surface, brown to dark brown ascospores with straight germ slit and dehiscent perispore with very conspicuous coil-like ornamentation. Although the phylogenetic trees (Figure 1 and Figure S1) show that *H. medogense* and *H. erythrostroma* are closely related, as well as similar to each other in stromatal morphology and KOH-extractable pigments, *H*. *erythrostroma* was originally described and illustrated by Miller (1933) from Florida, and can be distinguished from *H. medogense* by having smaller ascospores (6.5–9.5 × 3–4.5 µm) and a shorter spore-bearing portion of asci (40–50 µm). Ju and Rogers [12] reexamined the isotype of *H.* *erythrostroma* (GAM 2374) from the USA and other specimens from Brazil, French Guiana, Madagascar, Mexico, Papua New Guinea, and Puerto Rico, and found that the fungi has smaller ascospores ((7–)7.5–9.5 × 3–4.5 µm) with sigmoid germ slit spore-length and inconspicuous coil-like perispore ornamentation; the species was also reported in Guadeloupe (French West Indies) by Fournier et al. [10].

Notably, *Hypoxylon medogense* shows morphological similarities to *H. crocopeplum* Berk., M.A. Curtis and *H. laschii* Nitschke in stromatal morphology. *Hypoxylon crocopeplum* can be distinguished by obovoid to long tubular perithecia (0.1–0.3(–0.4) mm broad × 0.2–1.5 mm high), longer asci ((100–)120–205(–217) µm total length) and slightly larger ascospores ((9–)9.5–15(–17.5) × 4–7(–7.5) µm) with inconspicuous to conspicuous coil-like perispore ornamentation. *Hypoxylon laschii* has longer asci (165–190 µm total length) and smaller ascospores (8–10 × 3.5–4.5 µm) with no perspore ornamentation [12]. In the phylogenetic trees, *H. medogense* is distant from the two species.

**Figure 4:**
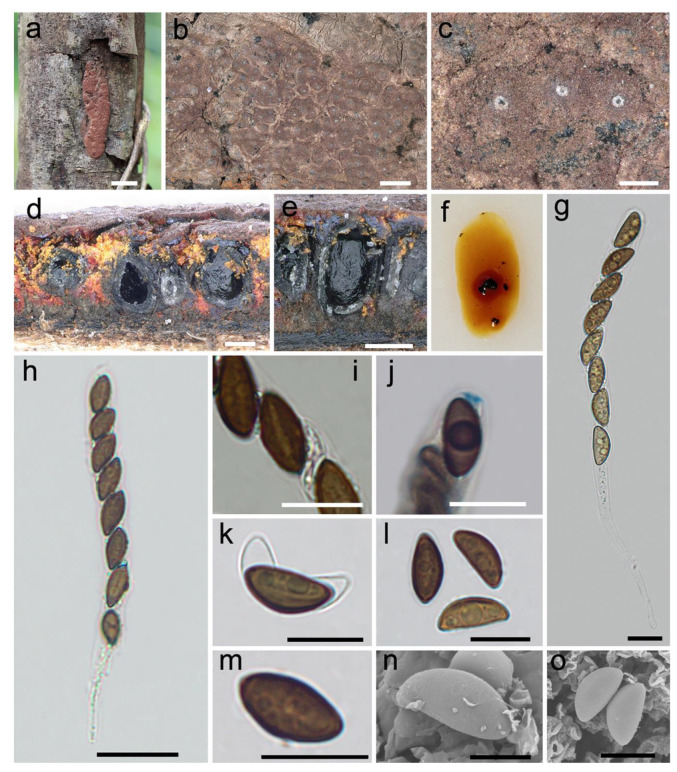
*Hypoxylon zangii* (holotype FCATAS 4029). (**a**) Stroma on the bark of dead wood. (**b**,**c**) Stromatal surface. (**d**,**e**) Stroma in vertical section showing perithecia and ostioles. (**f**) KOH-extractable pigments. (**g**,**h**) Asci in water. (**i**) Ascospores in water showing germ slit. (**j**) Apical apparatus in Melzer’s reagent. (**k**) Ascospore in 10% KOH. (**l**,**m**) Ascospores in water. (**n**,**o**) Ascospores under SEM. Scale bars: (**a**) = 1 cm; (**b**) = 1 mm; (**c**–**e**) = 200 µm; (**g**,**i**–**m**) = 10 µm; (**h**) = 20 µm; (**n**) = 5 µm; (**o**) = 8 µm.

***Hypoxylon zangii*** Hai X. Ma, Z.K. Song and Y. Li, sp. nov., Figure 4.

MycoBank: MB 843580

**Diagnosis.** Differs from *H. fendleri* and *H. retpela* in its smaller ascospores. Differs from *H. rubiginosum* in its stromatal granules and a subtropical distribution. Differs from *H. texense* in its stromatal KOH-extractable pigments and larger ascospores. Differs from *H. guilanense* in its stromatal morphology.

**Etymology.***Zangii* (Lat.): referring in honor to Chinese mycologist Dr. Zang Mu, who is also the author of “Field Records in the Mountains and Valleys: Discovery Journey to the Third Pole—Notes and Drawings of Zang Mu Scientific Expeditions”.

**Holotype.** CHINA: Tibet Autonomous Region, Medog County, Yarlung Zangbo River, the large bend of Linduo, 29°27′52″ N, 95°26′39″ E, alt. 781 m, saprobic on the bark of dead wood, 24 September 2021, Haixia Ma, Col. XZ29 (FCATAS 4029).

**Teleomorph.** Stromata effused-pulvinate, 1.2–4.1 cm long × 0.8–1 cm broad × 0.25–0.45 mm thick; with conspicuous perithecial mounds; surface livid red (56) and vinaceous (57); with orange or reddish orange granules immediately beneath the surface and between perithecia; yielding amber (47), fulvous (43) and sienna (8) KOH-extractable pigments; tissue below the perithecial layer inconspicuous, brown. Perithecia spherical, ovoid to obovoid, black, 0.2–0.4 mm broad × 0.3–0.5 mm high. Ostioles umbilicate, sometimes overlain with conspicuous white substance, opening lower than the stromatal surface. Asci cylindrical, eight-spored, uniseriate, 85–145 µm total length, the spore-bearing portion 65–92 µm long × 7.1–10.9 µm broad, and stipes 12–66 µm long, with amyloid apical apparatus bluing in Melzer’s reagent, discoid, 0.8–1.3 µm high × 2–2.9 µm broad. Ascospores light-brown to brown, unicellular, ellipsoid-inequilateral, with slightly acute to narrowly rounded ends, 10.9–14.6 × 4.8–6.4 µm (n = 60, M = 12.2 × 5.5 µm), with straight spore-length germ slit on the convex side; perispore dehiscent in 10% KOH, with inconspicuous coil-like ornamentation in SEM; epispore smooth.

**Additional specimens examined.** CHINA: Tibet Autonomous Region, Medog County, Yarlung Zangbo River, the larger bend of Linduo, 29°27′35″ N, 95°26′32″ E, alt. 780 m, saprobic on the bark of dead wood, 24 September 2021, Haixia Ma, Col. XZ319 (FCATAS 4319).

**Note.** The stromatal morphology of *H. zangii* is similar to *H. fendleri* Berk. ex Cooke, *H. retpela* Van der Gucht, Van der Veken and *H. rubiginosum*. However, *H. fendleri* differs by having slightly thicker stromata at 0.5–0.8 mm, smaller ascospores ((8–)9–12 × 4–5.5 µm) with sigmoid germ slit spore-length, while *H. retpela* has thicker stromata at 0.5–0.8 mm, and smaller ascospores ((9–)9.5–12 × 4.5–5 µm) with very conspicuous coil-like ornamentation [12]. *Hypoxylon rubiginosum* can also be distinguished by its yellowish-brown or brown stromatal granules, thicker stromata (0.5–1.2(–1.5) mm) and smaller ascospores ((8–)9–12 × 4–5.5 µm). In addition, H. *rubiginosum* prefers to distribute in the northern temperate region, while *H. zangii* was found in subtropical region [12,15,47]. These three species are distant from *H. zangii* in the phylogenetic trees (Figure 1).

*Hypoxylon zangii* clustered with *H. guilanense* and *H. texense* in a strong support clade in the phylogenetic trees. *Hypoxylon texense* shows morphological similarities to *H. zangii* with reddish-orange stromatal granules, but differs in having rust (39) to dark brick (86) instead of amber (47), fulvous (43) and sienna (8) KOH-extractable pigments, and smaller ascospores ((9–)9.5–12 × 4.5–5 µm) with straight to slightly sigmoid germ slit spore-length [37]. *Hypoxylon guilanense* differs from *H. zangii* in having hemispherical to pulvinate stromata with sienna (8), umber (9) to buff (45) surface colors, with conspicuous perithecial mounds, and slightly larger ascospores (12–15 × 5–6 µm) with conspicuous coil-like ornamentation [15].

        **Dichotomous key to *Hypoxylon* species from China**

             **and related species worldwide**

1. Ascospores nearly equilateral ............................................................................................. 2

1. Ascospores inequilateral ...................................................................................................... 8

2. Ostiolar barely to slightly higher than the stromatal surface ......................................... 3

2. Ostioles lower than the stromatal surface ......................................................................... 4

3. Perithecia spherical, (0.2–)0.3–0.4 mm broad .................................................. ***H*.** ***croceum***

3. Perithecia spherical to tubular, 0.3–0.6 mm broad × 0.4–0.8 mm high. ***H*.** ***parksianum***

4. Perispore dehiscent in 10% KOH ............................................................... ***H. hypomiltum***

4. Perispore indehiscent in 10% KOH .................................................................................... 5

5. Perithecia tubular to long tubular ....................................................................................... 6

5. Perithecia obovoid ................................................................................................................ 7

6. KOH-extractable pigments orange (7) ................................................... ***H. cinnabarinum***

6. KOH-extractable pigments greenish yellow (16), dull green (70), or dark green

(21) ...................................................................................................................... ***H. investiens***

7. Stromatal surface brown vinaceous (84), sepia (63), or chestnut (40); without apparent

KOH-extractable pigments or with dilute grayish sepia (106) to blackish 

pigments ........................................................................................................ ***H. dieckmannii***

7. Stromatal surface fawn (87) or umber (9); KOH-extractable pigments hazel

(88) ................................................................................................................... ***H. gilbertsonii***

8. Ostiolar barely to slightly higher than the stromatal surface ......................................... 9

8. Ostioles lower than the stromatal surface ....................................................................... 15

9. Perithecia tubular.................................................................................... ***H. lienhwacheense***

9. Perithecia spherical, ovoid to obovoid ............................................................................. 10

10. Stromatal granules black ............................................................................... ***H. hainanense***

10. Stromatal granules colored ................................................................................................ 11

11. Stromata glomerate; KOH-extractable pigments hazel (88) .................. ***H. lenormandii***

11. Stromata pulvinate; KOH-extractable pigments orange (7) ......................................... 12

12. Sigmoid germ slit ................................................………......................... ***H. erythrostroma***

12. Straight germ slit ................................................................................................................. 13

13. Perispore with very conspicuous coil-like ornamentation ....................... ***H. medogense***

13. Perispore smooth or with inconspicuous coil-like ornamentation .............................. 14

14. Stromata pulvinate to discoid, erumpent, usually encircled with ruptured plant tissue;

perithecia 0.2–0.4(–0.5) mm diam ............................................................ ***H. laschii***

14. Stromata pulvinate to effused-pulvinate, sometimes hemispherical, plane; perithecia 

0.1–0.2 mm diam ................................................................................................... ***H. rutilum***

15. Sigmoid germ slit .........................................………........................………………… 16

15. Straight or slightly sigmoid germ slit ............................................................................... 19

16. Perispore with conspicuous coil-like ornamentation ................... ***H*.** ***cyclobalanopsidis***

16. Perispore smooth or with inconspicuous coil-like ornamentation .............................. 17

17. Sigmoid germ slit much less than spore-length; stromata glomerate, with conspicuous

perithecial mounds; KOH-extractable pigments pure yellow (14) with citrine (13) tone, 

greenish olivaceous (90), or orange (7) ............................. ***H. musceum***

17. Sigmoid germ slit spore-length; stromata pulvinate or effused-pulvinate, with inconsp

icuous to conspicuous perithecial mounds; KOH-extractable pigments with other

colors ..................……................................................................................................. 18

18. KOH-extractable pigments orange (7) ..................……..................................... ***H. fendleri***

18. KOH-extractable pigments vinaceous purple (101) ............…..................... ***H. fuscoides***

19. Perispore infrequently dehiscent in 10% KOH .................…….....................……......... 20

19. Perispore dehiscent in 10% KOH .................…….........................…................................ 22

20. Stromata saprobic on surface of dead bamboo ................................... ***H.*** ***wuzhishanense***

20. Stromata saprobic on the bark of dicot wood ................................................................. 21

21. Ascospores light-brown to brown, 8.2–10.5 × 4.1–5.5 µm, with straight germslit spore-

length ....................................................................................................... ***H. damuense***

21. Ascospores brown to dark brown, (10–)10.5–11.5(–12.5) × 5–6.5 µm, with straight germ

slit slightly less than spore-length .............................................................. ***H. dengii***

22. Perispore with conspicuous coil-like ornamentation .................................................... 23

22. Perispore smooth or with inconspicuous coil-like ornamentation .............................. 28

23. Stromata pulvinate to effused-pulvinate ......................................................................... 24

23. Stromata glomerate or hemispherical .............................................................................. 25

24. Perithecia tubular to long tubular or obovoid, 0.2–0.3 mm broad × 0.6–0.9 mm high;

ascospores light brown to dark brown, 10.3–13.6 × (4.2–) 4.7–6.1 μm, with conspicuous

straight germ slit .................................................................... ***H. jianfengense***

24. Perithecia spherical to obovoid, 0.2–0.3 mm broad × 0.2–0.5 mm high; ascospores

brown to dark brown, (9–)9.5–12 × 4.5–5 μm, with straight to slightly sigmoid germ

slit ............................................................................................................................. ***H. retpela***

25. KOH-extractable pigments orange (7) ............................................................................. 26

25. KOH-extractable pigments with other colors ................................................................. 27

26. Stromata glomerate to pulvinate; stromatal granules dull yellow

or rust ............................................................................................................. ***H. baihualingense***

26. Stromata hemispherical to pulvinate; stromatal granules scarlet (5) to orange

(7) ....................................................................................................................... ***H. guilanense***

27. Stromatal granules pale brown to dull reddish-brown; KOH-extractable pigments pale

luteous (11), honey (60) and ochreous (44); apical apparatus highly reduced or lacking,

not bluing in Melzer’s reagent; ascospores light-brown to brown, with slightly broad

rounded ends, 8–10.6(–11.1) × 4.1–6.3(–7.1) µm ... ***H. chrysalidosporum***

27. Stromatal granules dull reddish-brown to blackish; KOH-extractable pigments

 isabelline (65) or amber (47); apical apparatus bluing in Melzer’s reagent; ascospores

brown to dark brown, with narrowly rounded ends, 9.5–13(–14.5) × 4.5–6.5

µm ........................................................................................................................... ***H. duranii***

28. KOH-extractable pigments greenish to olivaceous ........................................................ 29

28. KOH-extractable pigments with other colors ................................................................. 33

29. Stromata pulvinate to effused-pulvinate ......................................................................... 30

29. Stromata glomerate or hemispherical .............................................................................. 31

30. Ascospores brown to dark brown, 8.5–13.5 × 4–6 μm .......................... ***H*. *anthochroum***

30. Ascospores light brown to brown, 5.5–8 × 2.5–3.5 μm ........................... ***H. brevisporum***

31. Apical apparatus highly reduced or lacking, not bluing in Melzer’s rea

gent .................................................................................................................. ***H. notatum***

31. Apical apparatus bluing in Melzer’s reagent .................................................................. 32

32. Perithecia spherical to obovoid, 0.1–0.3(–0.4) mm broad × 0.2–0.5 mm high; slightly

sigmoid germ slit ................................................................................................... ***H*. *fuscum***

32. Perithecia long tubular, 0.3–0.6 mm broad × (0.6–)0.8–2 mm high; straight germ

slit ................................................................................................................. ***H. placentiforme***

33. Stromata hemispherical ...................................................................................................... 34

33. Stromata pulvinate to effused-pulvinate ......................................................................... 37

34. Perithecia long tubular .......................................................................... ***H. haematostroma***

34. Perithecia spherical to obovoid ......................................................................................... 35

35. KOH-extractable pigments amber (47) with greenish yellow (16) tone, or greenish 

yellow (16) with citrine (13) tone ................................................................ ***H*. *perforatum***

35. KOH-extractable pigments orange (7) ............................................................................. 36

36. Apical apparatus bluing in Melzer’s reagent, 0.8–1.2 μm high × 2.2–2.8 μm broad;

ascospores (10.5–)11–15 × 5–6.5(–7) μm ....................................................... ***H. fragiforme***

36. Apical apparatus bluing in Melzer’s reagent, 0.4–0.8 μm high × 1.2–2 μm broad;

ascospores 7–9.5(–10) × 3–4.5 μm ................................................................. ***H. howeanum***

37. Perithecia tubular ................................................................................................................ 38

37. Perithecia spherical to obovoid ......................................................................................... 42

38. Stromatal granules black; KOH-extractable pigments dark livid (80) .... ***H. lividicolor***

38. Stromatal granules colored; KOH-extractable pigments with other colors ............... 39

39. KOH-extractable pigments pure yellow (14) or amber (47) ......................... ***H. trugodes***

39. KOH-extractable pigments orange (7) ............................................................................. 40

40. Apical apparatus bluing in Melzer’s reagent, 0.2–0.5 μm high × 1–1.5 μm

broad ................................................................................................................... ***H. jecorinum***

40. Apical apparatus lightly bluing or bluing in Melzer’s reagent, more than 1.5 μm

broad ..................................................................................................................................... 41

41. Perithecia spherical, obovoid to long tubular, up to 1.5 mm high; ascospores (9–)9.5

–15(–17.5) × 4–7(–7.5) μm; *Virgariella*-like conidiogenous structure

 ........................................................................................................ ***H*. *crocopeplum***

41. Perithecia obovoid to tubular, up to 0.7 mm high; ascospores 7–11 × 3.5–5 μm; 

*Nodulisporium*-like conidiogenous structure ................................................ ***H. subgilvum***

42. Stromata saprobic on dead bamboo .......................................................... ***H. pilgerianum***

42. Stromata saprobic on dicot wood ..................................................................................... 43

43. Ascospores 15.5–22.9(–23.6) × 7.3–10.6 μm ....................................................... ***H. larissae***

43. Ascospores length less than 15 µm ................................................................................... 44

44. Perithecia subglobose, 0.5–0.7 mm broad; straight or slightly sigmoid germ slit nearly

spore-length ...................................................................................... ***H. wujiangense***

44. Perithecia less than 0.5 mm broad; straight germ slit spore-length ............................. 45

45. Stromatal granules orange or reddish orange; ascospores light-brown ..................... 46

45. Stromatal granules yellowish-brown or dull purplish-brown; ascospores dark 

brown .................................................................................................................................... 47

46. KOH-extractable pigments rust (39) to dark brick (86); ascospore (8.7–)9.1–10.8(–11.5)

× (4.0–)4.5–5.4 μm .................................................................................................. ***H. texense***

46. KOH-extractable pigments amber (47), fulvous (43) and sienna (8); ascospore 10.9–14.6

× 4.8–6.4 µm ............................................................................................. ***H. zangii***

47. Stromatal granules yellowish-brown or brown; perithecia 0.2–0.5 mm broad × 0.3–0.6

mm high; smooth or with inconspicuous coil-like ornamentation perispore; *Periconiella*-

like conidiogenous structure .................................................. ***H. rubiginosum***

47. Stromatal granules dull purplish-brown; perithecia 0.1–0.2 mm broad × 0.2–0.3 mm

high; smooth perispore; *Nodulisporium*-like conidiogenous structure

 .............................................................................................. ***H. vinosopulvinatum***

## 4. Discussion

In the present study, three species of *Hypoxylon* from Medog in China, *H. damuense*, *H. medogense*, and *H. zangii*, are described as new species based on molecular analyses and morphological features. Phylogenetic analyses on the species of *Hypoxylon* presented confirmed that *Hypoxylon* is a polyphyletic genus. The species analyzed appeared mainly distributed in six separate clades (except *H. papillatum* Ellis, Everh. and *H. dieckmannii* Theiss.). *Hypoxylon damuense* and *H. zangii* were clearly separated from other sampled species of *Hypoxylon* and from each other in the clade H2, and *H. medogense* was included in clade H3 containing *H. fragiforme* (Pers.) J. Kickx f., the type species of the genus. The phylogenetic tree shows that the classification of *Hypoxylon* is confusing. It did not suggest any apparent correlation in morphological features with the distribution of species in the phylogenetic trees. Therefore, more collections, more gene sequences and new taxonomic features, as well as the application of polyphasic taxonomic approaches based on morphological (sexual and asexual), chemotaxonomic, and phylogenetic data of this genus are needed in the further studies. Previously numerous new species have been found in Southwest China [49,50], and present paper confirmed that more known fungal species in the area.

## Figures and Tables

**Table 1:** GenBank accession numbers of sequences used in the multi-gene phylogenetic analyses. T and ET represent holotype and epitype specimens, respectively. Species in bold were derived from this study. N/A: not available.

Species Name	Specimen No.	Locality	GenBank Accession No.					References
			ITS	LSU	RPB2	β-Tubulin	Status	
*Annulohypoxylon annulatum*	CBS 140775	USA	KU604559	KY610418	KY624263	KX376353	ET	[2,11,25]
*A*. *moriforme*	CBS 123579	Martinique	KX376321	KY610425	KY624289	KX271261		[25]
*A*. *truncatum*	CBS 140778	USA	KX376329	KY610419	KY624277	KX376352	ET	[2,25]
*Daldinia dennisii*	CBS 114741	Australia	JX658477	KY610435	KY624244	KC977262	T	[2,9,34]
*D*. *petriniae*	MUCL 49214	Austria	JX658512	KY610439	KY624248	KC977261	ET	[2,9,34]
*Hypomontagnella barbarensis*	STMA 14081	Argentina	MK131720	MK131718	MK135891	MK135893	T	[35]
*Hypom*. *monticulosa*	MUCL 54604	French Guiana	KY610404	KY610487	KY624305	KX271273	ET	[2]
*Hypom*. *submonticulosa*	CBS 115280	France	KC968923	KY610457	KY624226	KC977267		[2,9]
*Hypoxylon addis*	MUCL 52797	Ethiopia	KC968931	N/A	N/A	KC977287	T	[9]
*H*. *anthochroum*	YMJ 9	Mexico	JN660819	N/A	N/A	AY951703		[13]
*H*. *aveirense*	CMG 29	Portugal	MN053021	N/A	N/A	MN066636	T	[36]
*H.* *baihualingense*	FCATAS 477	China	MG490190	N/A	N/A	MH790276	T	[18]
*H.* *baruense*	UCH 9545	Panama	MN056428	N/A	N/A	MK908142		[32]
*H*. *begae*	YMJ 215	USA	JN660820	N/A	N/A	AY951704		[13]
*H*. *bellicolor*	UCH 9543	Panama	MN056425	N/A	N/A	MK908139		[32]
*H*. *brevisporum*	YMJ 36	Puerto Rico	JN660821	N/A	N/A	AY951705		[13]
*H*. *carneum*	MUCL 54177	France	KY610400	KY610480	KY624297	KX271270		[2]
*H*. *cercidicola*	CBS 119009	France	KC968908	KY610444	KY624254	KX271270		[2,9]
*H* *. chrysalidosporum*	FCATAS 2710	China	OL467294	OL615106	OL584222	OL584229	T	[20]
*H*. *crocopeplum*	CBS 119004	France	KC968907	KY610445	KY624255	KC977268		[2]
*H* *. cyclobalanopsidis*	FCATAS 2714	China	OL467298	OL615108	OL584225	OL584232	T	[20]
***H. damuense***	**FCATAS** **4207**	**China**	**ON075427**	**ON075433**	**ON093251**	**ON093245**	**T**	**This study**
***H. damuense***	**FCATAS** **4321**	**China**	**ON075428**	**ON075434**	**ON093252**	**ON093246**		**This study**
*H*. *dieckmannii*	YMJ 89041203	China	JN979413	N/A	N/A	AY951713		[13]
*H*. *duranii*	YMJ 85	China	JN979414	N/A	N/A	AY951714		[13]
*H*. *erythrostroma*	YMJ 90080602	China	JN979416	N/A	N/A	AY951716		[13]
*H*. *eurasiaticum*	MUCL 57720	Iran	MW367851	N/A	MW373852	MW373861		[37]
*H*. *fendleri*	DSM 107927	USA	MK287533	MK287545	MK287558	MK287571		[38]
*H*. *ferrugineum*	CBS 141259	Austria	KX090079	N/A	N/A	KX090080		[23]
*H*. *fragiforme*	MUCL 51264	Germany	KM186294	KM186295	KM186296	KM186293	ET	[38]
*H*. *fraxinophilum*	MUCL 54176	France	KC968938	N/A	N/A	KC977301	ET	[9]
*H*. *fulvosulphureum*	MFLUCC 13-0589	Thailand	KP401576	N/A	N/A	KP401584	T	[39]
*H*. *fuscum*	CBS 113049	France	KY610401	KY610482	KY624299	KX271271	ET	[2]
*H*. *griseobrunneum*	CBS 331.73	India	KY610402	MH872399	KY624300	KC977303	T	[2,9,40]
*H*. *guilanense*	MUCL 57726	Iran	MT214997	MT214992	MT212235	MT212239	T	[15]
*H*. *haematostroma*	MUCL 53301	Martinique	KC968911	KY610484	KY624301	KC977291	ET	[35]
*H*. *hinnuleum*	MUCL 3621	USA	MK287537	MK287549	MK287562	MK287575	T	[38]
*H*. *howeanum*	MUCL 47599	Germany	AM749928	KY610448	KY624258	KC977277		[2,9,41]
*H*. *hypomiltum*	MUCL 51845	Guadeloupe	KY610403	KY610449	KY624302	KX271249		[2]
*H*. *invadens*	MUCL 51475	France	MT809133	MT809132	MT813037	MT813038	T	[42]
*H*. *investiens*	CBS 118183	Malaysia	KC968925	KY610450	KY624259	KC977270		[2,9]
*H*. *isabellinum*	STMA 10247	Martinique	KC968935	N/A	N/A	KC977295	T	[9]
*H. jecorinum*	YMJ 39	Mexico	JN979429	N/A	N/A	AY951731		[13]
*H.* *jianfengense*	FACATAS845	China	MW984546	MZ029707	MZ047260	MZ047264	T	[21]
*H. larissae*	FACATAS844	China	MW984548	MZ029706	MZ047258	MZ047262	T	[21]
*H*. *lateripigmentum*	MUCL 53304	Martinique	KC968933	KY610486	KY624304	KC977290	T	[2,9]
*H*. *lenormandii*	CBS 135869	Cameroon	KY610390	KY610453	KY624262	KM610295		[2,43]
*H*. *liviae*	CBS 115282	Norway	NR155154	N/A	N/A	KC977265	ET	[9]
*H*. *lividicolor*	YMJ 70	China	JN979432	N/A	N/A	AY951734		[13]
*H*. *lividipigmentum*	YMJ 233	Mexico	JN979433	N/A	N/A	AY951735		[13]
*H*. *macrosporum*	YMJ 47	Canada	JN979434	N/A	N/A	AY951736		[13]
***H. medogense***	**FCATAS** **4061**	**China**	**ON075425**	**ON075431**	**ON093249**	**ON093243**	**T**	**This study**
***H. medogense***	**FCATAS** **4320**	**China**	**ON075426**	**ON075432**	**ON093250**	**ON093244**		**This study**
*H*. *musceum*	MUCL 53765	Guadeloupe	KC968926	KY610488	KY624306	KC977280		[2,9]
*H*. *notatum*	YMJ 250	USA	JQ009305	N/A	N/A	AY951739		[13]
*H*. *olivaceopigmentum*	DSM 10792	USA	MK287530	MK287542	MK287555	MK287568	T	[38]
*H*. *papillatum*	ATCC 58729	USA	NR155153	KY610454	KY624223	KC977258	T	[2,9]
*H*. *perforatum*	CBS 115281	France	KY610391	KY610455	KY624224	KX271250		[2]
*H*. *petriniae*	CBS 114746	France	NR155185	KY610491	KY624279	KX271274	T	[2]
*H*. *pilgerianum*	STMA 13455	Martinique	KY610412	N/A	KY624308	KY624315		[2]
*H*. *porphyreum*	CBS 119022	France	KC968921	KY610456	KY624225	KC977264		[2,9]
*H*. *pseudofendleri*	MFLUCC 11-0639	Thailand	KU940156	KU863144	N/A	N/A		[44]
*H*. *pseudofuscum*	18264	Germany	MW367857	MW367848	MW373858	MW373867	T	[37]
*H*. *pulicicidum*	CBS 122622	Martinique	JX183075	KY610492	KY624280	JX183072	T	[2,45]
*H*. *rickii*	MUCL 53309	Martinique	KC968932	KY610416	KY624281	KC977288	ET	[2]
*H*. *rubiginosum*	MUCL 52887	Germany	KC477232	KY610469	KY624266	KY624311	ET	[2,46]
*H*. *rutilum*	YMJ 181	France	N/A	N/A	N/A	AY951752		[13]
*H*. *samuelsii*	MUCL 51843	Guadeloupe	KC968916	KY610466	KY624269	KC977286	ET	[2,9]
*H*. *shearii*	YMJ 29	Mexico	EF026142	N/A	N/A	AY951753		[13]
*H*. *spegazzinianum*	STMA 14082	Argentina	KU604573	N/A	N/A	KU604582	T	[11]
*H*. *sporistriatatunicum*	UCH 9542	Panama	MN056426	N/A	N/A	MK908140	T	[32]
*H*. *subgilvum*	YMJ 88113007	China	JQ009315	N/A	N/A	AY951755		[13]
*H*. *sublenormandii*	JF 13026	Sri Lanka	KM610291	N/A	N/A	KM610303	T	[43]
*H*. *texense*	DSM 107933	USA	MK287536	MK287548	MK287561	MK287574	T	[38]
*H*. *ticinense*	CBS 115271	France	JQ009317	KY610471	KY624272	AY951757		[2,13]
*H*. *trugodes*	MUCL 54794	Sri Lanka	KF234422	NG066380	KY624282	KF300548	ET	[2,9]
*H*. *ulmophilum*	YMJ 350	Russia	JQ009320	N/A	N/A	AY951760		[13]
*H*. *vogesiacum*	CBS 115273	France	KC968920	KY610417	KY624283	KX271275		[2]
*H. wujiangense*	GMBC0213	China	MT568854	MT568853	MT585802	MT572481	T	[19]
*H*. *wuzhishanense*	FCATAS2708	China	OL467292	OL615104	OL584220	OL584227	T	[20]
***H. zangii***	**FCATAS** **4029**	**China**	**ON075423**	**ON075429**	**ON093247**	**ON093241**	**T**	**This study**
***H. zangii***	**FCATAS** **4319**	**China**	**ON075424**	**ON075430**	**ON093248**	**ON093242**		**This study**
*Jackrogersella cohaerens*	CBS 119126	Germany	KY610396	KY610497	KY624270	KY624314		[2]
*J*. *multiformis*	CBS 119016	Germany	KC477234	KY610473	KY624290	KX271262	ET	[2,9]
*Pyrenopolyporus hunteri*	MUCL 52673	Ivory Coast	KY610421	KY610472	KY624309	KU159530	ET	[2,25]
*P*. *laminosus*	MUCL 53305	Martinique	KC968934	KY610485	KY624303	KC977292	T	[2,9]
*P*. *nicaraguensis*	CBS 117739	Burkina Faso	AM749922	KY610489	KY624307	KC977272		[2,9,41]
*Rhopalostroma angolense*	CBS 126414	Ivory Coas	KY610420	KY610459	KY624228	KX271277		[2]
*Thamnomyces dendroidea*	CBS 123578	French Guiana	FN428831	KY610467	KY624232	KY624313	T	[2,47]
*Xylaria hypoxylon*	CBS 122620	Sweden	KY610407	KY610495	KY624231	KX271279	ET	[2]
*Biscogniauxia nummularia*	MUCL 51395	France	KY610382	KY610427	KY624236	KX271241		[2]

## Data Availability

All newly generated sequences were deposited in GenBank (https://www.ncbi.nlm.nih.gov/genbank/, accessed on 15 March 2022; Table 1). All new taxa were deposited in MycoBank (https://www.mycobank.org/, accessed on 12 March 2022; MycoBank identifiers follow new taxa).

## Supplementary Materials

The following supporting information can be downloaded at: https://www.mdpi.com/article/10.3390/jof8050500/s1, Figure S1: ML phylogram inferred from ITS-TUB2 sequences. ML bootstrap support (BS) ≥ 50% and Bayesian posterior probabilities (PP) ≥ 0.95 are labelled above or below the respective branches (BS/PP). Species in bold were sequenced in the this study.
