# Supplementary material for: Three New Species of Hypoxylon (Xylariales, Ascomycota) on a Multigene Phylogeny from Medog in Southwest China
Source: J Fungi (Basel). 2022 May 11;8(5):500. doi: 10.3390/jof8050500 (PMC9146989; doi:10.3390/jof8050500)
Supplement: Supplementary file 1 [file jof-08-00500-s001.zip › jof-1696637-supplementary.pdf]

Journal of *Fungi*

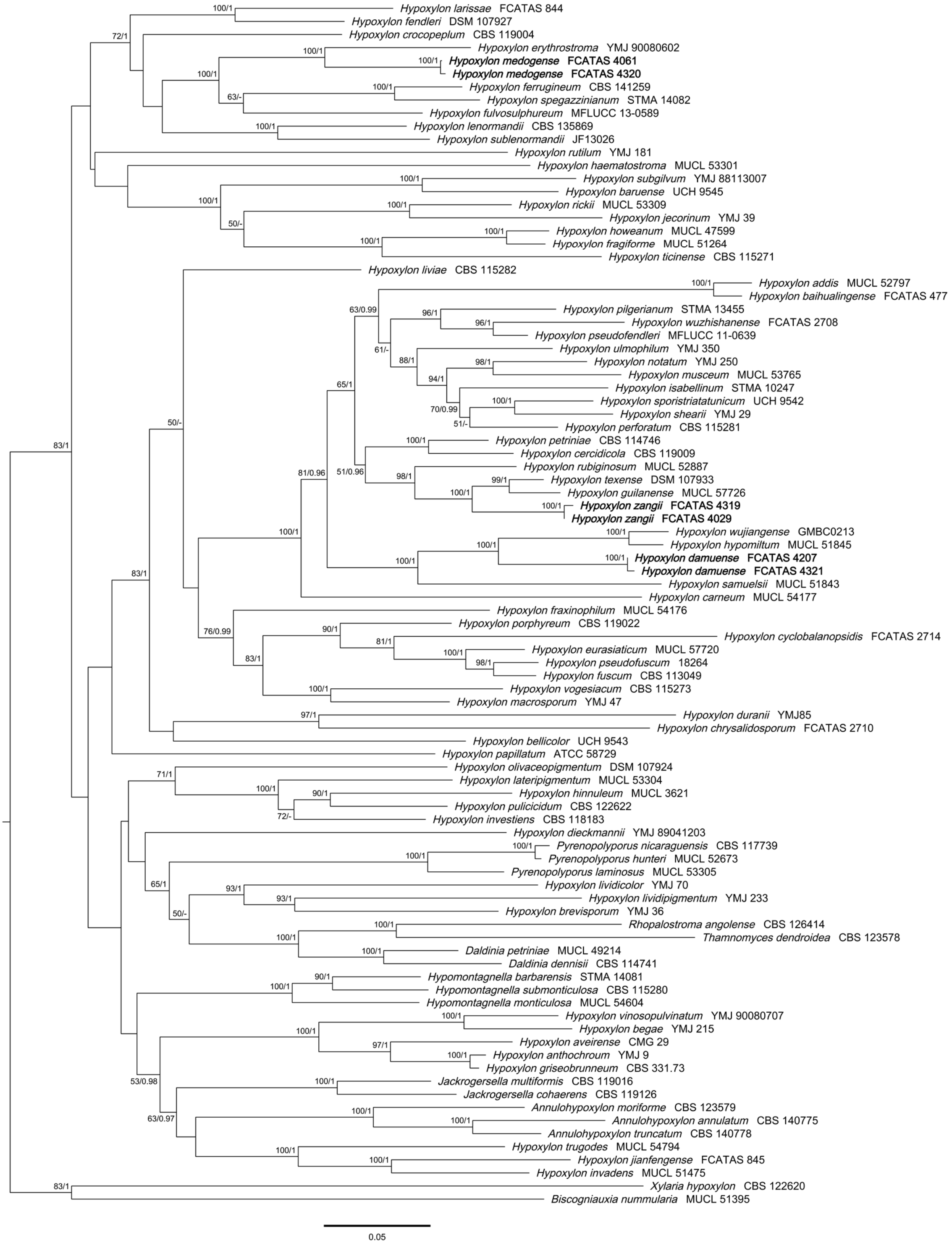

Figure S1. ML phylogram inferred from ITS-TUB2 sequences. ML bootstrap support (BS) ≥ 50% and Bayesian posterior probabilities (PP) ≥ 0.95 are labelled above or below the respective branches (BS/PP). Species in bold were sequenced in the this study.
